# The future of animal science departments

**DOI:** 10.1093/af/vfaa020

**Published:** 2020-07-23

**Authors:** Kristen A Johnson

**Affiliations:** Department of Animal Science, Washington University, Pullman, WA

“Education is our passport to the future, for tomorrow belongs only to the people who prepare for it today.”

Malcolm X

This issue of *Animal Frontiers* is envisioned to be a reflection on the future of animal or veterinary science departments in institutions of higher education. The challenges associated with funding for animal research, the maintenance of research and teaching infrastructure, finding and training qualified students for future careers, and educating administrators about the costs and challenges associated with animal research and teaching programs are known across the world. How animal science departments address these issues and others will determine the future. The exercise of imagining the future is critical to shaping it and this issue of *Animal Frontiers* (Figure 1) encompasses the thoughts about the future from around the world.

**Figure 1.**
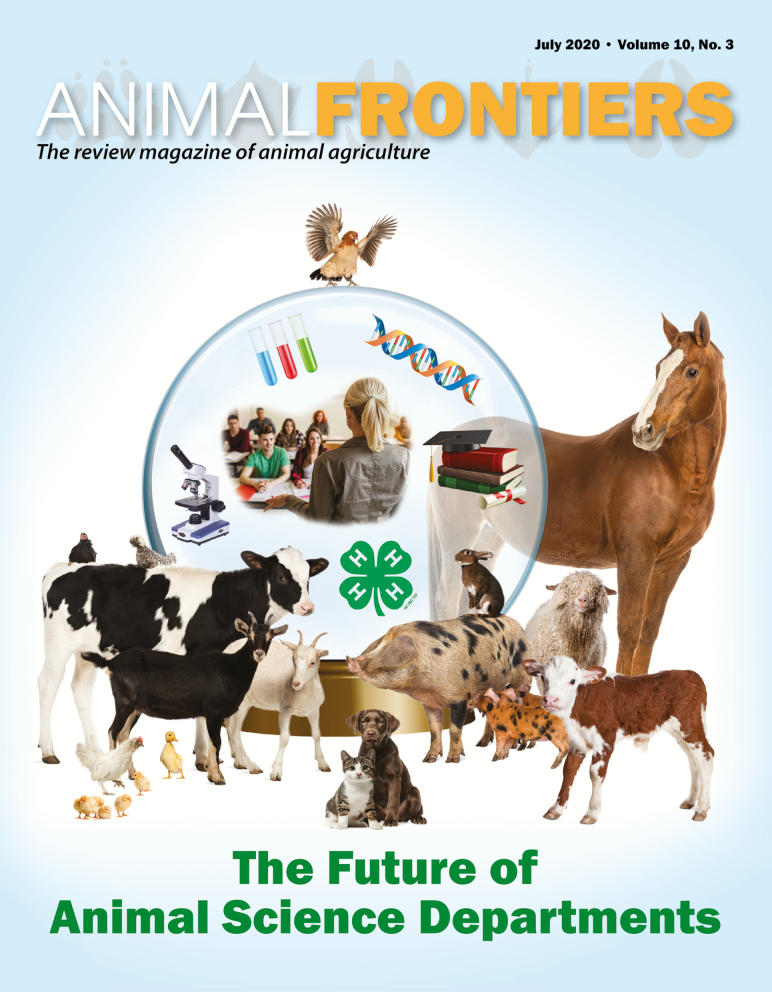
The future of animal science departments.

Klaus Schwab (2015) first introduced the term the “Fourth Industrial Revolution” to describe the current technological revolution that encompasses the developments in artificial intelligence, biotechnology, genomics, robotics, and other revolutionizing technologies (Schwab, 2015). In 2016, the World Economic Forum (WEF) published a Global Insight Report entitled “The Future of Jobs: Employment, Skills and Workforce Strategy for the Fourth Industrial Revolution.” This publication detailed that the workforce in the technological revolution will need skills including enhanced cognitive abilities (logical reasoning, creativity, and cognitive flexibility) and complex problem-solving skills (critical thinking and decision-making). Social skills (people management, emotional intelligence, and service orientation) and content and process skills were also identified as critical. Other recommendations were to redesign disciplinary education to merge training of the humanities and sciences and create people who are capable of multidisciplinary thinking. This change in emphasis would improve training in critical thinking and problem solving and allow for continual renewal of the workforce’s skills (Schwab and Samans, 2016). These thoughts are echoed by Dr Stephen Gavazzi (Gravazzi, 2020) who challenges land grant universities in the United States to return to the founding land grant principles and to recognize the importance of a community-based mission for the 21st century. His ideas provide an excellent foundation for articles from Argentina, China, Italy, and South Africa that describe the future of animal science research and teaching programs in these countries.

Workforce training is discussed in the article by Dr Gustavo Jaurena and Dr Maria Boveri (Jaurena and Boveri, 2020) from Argentina. They highlight the need for the incorporation of multidisciplinary training and thinking in the animal sciences curriculum and describe opportunities to embrace technology in information transfer in outreach and teaching. Rulien Grobler and her colleagues (Grobler et al., 2020) describe the dynamic animal sciences programs in South Africa and identify the need for flexibility and embracing technologies in training future animal scientists. The impact of COVID-19 and the need to embrace technology has reinforced the points made by Grobler et al. (2020). Faculty across the world have been forced to learn and incorporate online teaching with only a few days or weeks to prepare. A perspective from some faculty experiencing this rapid, radical change identifies some ideas and challenges for the future is included (Radcliffe et al., 2020). Much has been learned from the COVID-19 pandemic that will be useful and could revolutionize how information is shared: potentially meeting some of the foundational principles Gavazzi (2020) discusses.

The WEF publication (2016) also suggests public-private and cross industry partnerships as change agents and mechanisms by which the talent pool could be increased (Schwab and Samans, 2016). These same themes are found in this issue from contributions across the world. Baldi et al. (2020) describes the growing importance of public-private relationships in research and training in Italy. Animal science research and teaching is also becoming enriched by public-private partnerships in China. Dr. Jingdong Yin and Dr. Zhengpeng Zhu (Yin and Zhu, 2020) highlight the need for these relationships to ensure students are well-trained for the developing, high demand careers in China which require critical thinking skills and multidisciplinary approaches to animal production. As new methods for teaching are developed and used to meet the needs and careers of the future, animal and veterinary sciences must insure the effectiveness of the educational program offered. Accreditation is one way in which animal science programs can identify and address their strengths and weaknesses and assure a common set of learning goals are obtained. One such program is described by Benson et al. (2020). The principles described in this article provide a process for accreditation of animal science programs.

The similarity of challenges and needs for animal and veterinary training programs identified by the authors in this issue is both striking and comforting. Sharing ideas, successes, failures and preparations for the future such as those in this issue is critical. The talent, creativity and dedication across the global animal science community makes the future of animal science departments bright.

In the middle of preparing this issue of *Animal Frontiers* for publication, the SARS-Cov-2 virus made many issues for the future take a back seat to other compelling challenges faced by the authors. A primary challenge included moving face-to-face teaching and outreach to on-line platforms immediately, research programs were temporarily curtailed or downsized and people remained in their homes. We are very grateful to the authors who persevered in the submission and review of these articles despite the personal and professional difficulties they faced in their countries and in their personal lives.

## About the Author

Kristen Johnson is Professor in Animal Sciences at Washington State University, Pullman, WA, where she currently serves as Interim Chair, Department of Animal Sciences. She conducts research in beef cattle energetics and has applied this work to examine basic and applied issues to improve the efficiency and environmental sustainability of ruminant production. She also teaches graduate and undergraduate courses and mentors graduate students in animal sciences.
